# *Epilepsia Open*—August 2025 Announcements

**DOI:** 10.1002/epi4.70120

**Published:** 2025-08-19

## ILAE CONGRESSES

XVIII Workshop on Neurobiology of Epilepsy (WONOEP 2025)

25–29 August 2025

Portugal

36th International Epilepsy Congress

30 August–3 September 2025

Lisbon, Portugal

Neuroimaging in Epilepsy

25–26 September 2025

Genetic basis of developmental and epileptic encephalopathies workshop for the Eastern Mediterranean Epilepsy Health Care Providers

1–2 October 2025

Sfax, Tunisia

Visiting teacher program – Tunisia

6–8 November 2025

Sfax, Tunisia

Visiting teacher program

13–15 November 2025

Rabat, Morocco

Epilepsy in the elderly: at the crossroads of brain aging, neurodegeneration, and functional networks

21–22 November 2025

Marrakech, Morocco

## 2026

15th ILAE School on pre‐surgical evaluation for epilepsy and epilepsy surgery

19–23 January 2026

Brno, Czech Republic

19th Latin American Summer School on Epilepsy

23–28 February 2026

Guadalajara, Mexico

8th ILAE School on EEG in the First Year of Life

30 March–2 April 2026

Cambridge, UK

5th International Summer School of Neuropsychology

19–24 April 2026

Picardy, France

XIV Congreso Latinoamericano de Epilepsia

16–18 May 2026

Lima, Peru

1º Curso Latinoamericano de EEG Pediátrico

6–9 August 2026

TBC

16th European Epilepsy Congress

5–9 September 2026

Athens, Greece

16th Asian & Oceanian Epilepsy Congress

19–22 November 2026

Kuala Lumpur, Malaysia

## 2027

37th International Epilepsy Congress

28 August–1 September 2027

Amsterdam, Netherlands

## ILAE WEBINARS

Crises prolongées : premiers secours extrahospitaliers

12 August 2025

Paroxysmal Non‐Epileptic Events from Neonates to Children

22 August 2025

VNS and Epilepsy

11 September 2025

How to interpret genetic data: Germline and somatic mutations of GATOR1 genes and focal epilepsy

25 September 2025

How to manage ketogenic diet therapies for refractory status epilepticus

16 October 2025

How to use AI in clinical EEG interpretation

23 October 2025

ILAE e‐Forum: the role of digital technologies in the management of epilepsy

4 November 2025

How to interpret genetic data in SCN1A‐related disorders

27 November 2025

ILAE e‐Forum: EEG: when, why and how?

12 December 2025

## OTHER CONGRESSES

5th International Congress on Mobile Health and Digital Technology in Epilepsy

4–6 September 2025

Copenhagen, Denmark

19th European Congress for Clinical Neurophysiology

9–12 September 2025

London, UK

13th Philippine League Against Epilepsy Biennial National Epilepsy Congress

10–12 September 2025

Manila, Philippines

Epilepsy Center 2025 Symposia

10–13 September 2025

Cleveland, Ohio, USA

ILAE British Young Epilepsy Section (YES) Early Career Building Day

14 September 2025

Bournemouth, UK

ILAE British Branch 2025 Annual Scientific Conference

15–17 September 2025

Bournemouth, UK

9th Global Symposium on Ketogenic Therapies

17–19 September 2025

Paris, France

18th Baltic Sea Summer School on Epilepsy

17–26 September 2025

Online

IEL Annual Epilepsy Expert Day 2025

19 September 2025

Dublin, Ireland

Genetic epilepsies and other neuronal ion channel disorders: Mechanisms and therapeutic perspectives

23–25 September 2025

Tübingen, Germany

19th Panhellenic Congress of Epilepsy

25–28 September 2025

Heraklion, Crete, Greece

7th Bologna EPIPED‐EEG Course: reflex epilepsies: mechanisms and electro‐clinical manifestations

27 September–1 October 2025

Bologna, Italy

58th Annual Meeting of the Japan Epilepsy Society

2–4 October 2025

Utsunomiya, Japan

Defining the Landscape of Late‐Onset Unexplained Epilepsy

15–17 October 2025

South Casco, Maine, USA

3rd Annual Conference of the African Neurophysiological Society

23–25 October 2025

Accra, Ghana

2025 CLAE Annual Scientific Meeting

24–26 October 2025

Calgary, Alberta, Canada

Epilepsy Society of Australia 39th Annual Scientific Meeting

5–7 November 2025

Perth, Australia

3rd Advanced Course on the Pharmacological Treatment of Drug Resistant Epilepsies

7–9 November 2025

Palma de Mallorca, Spain

42nd International Conference on Advances in Psychiatry and Mental Health

10–11 November 2025

Dubai, UAE

In Search of Lost Time 6

12–14 November 2025

Rome, Italy

19th Asian Epilepsy Surgery Congress

21–23 November 2025

Pak Shek Kok, Hong Kong SAR

The 2025 ILAE British Branch Clinical Epilepsy 1‐Day Course for Doctors in Training & Others

22 November 2025

Birmingham, UK

Encephalitis 2025

3–4 December 2025

London, UK & Online

British Neurosurgical Research Group Meeting 2025

8–9 December 2025

London, England

## 2026

4th International Artificial Intelligence in Epilepsy and Neurological Disorders Conference

16–19 March 2026

San Juan, Puerto Rico

18th Eilat Conference on New Antiepileptic Drugs and Devices

3–6 May 2026

Madrid, Spain

64th Annual Meeting of the German Society for Epileptology

10–13 June 2026

Würzburg, Germany

